# Genomic differences between sequence types 1 and 104 of *Streptococcus suis* Serotype 2

**DOI:** 10.7717/peerj.14144

**Published:** 2022-10-06

**Authors:** Anusak Kerdsin, Dan Takeuchi, Yukihiro Akeda, Shota Nakamura, Marcelo Gottschalk, Kazunori Oishi

**Affiliations:** 1 Faculty of Public Health, Kasetsart University Chalermphrakiat Sakon Nakhon Province Campus, Sakon Nakhon, Thailand; 2 Research Institute for Microbial Diseases, Osaka University, Osaka, Japan; 3 Division of Infection Control and Prevention, Graduate School of Medicine, Osaka University, Osaka, Japan; 4 Department of Bacteriology I, National Institute of Infectious Diseases, Tokyo, Japan; 5 Genome Information Research Center, Research Institute for Microbial Diseases, Osaka University, Osaka, Japan; 6 Faculty of Veterinary Medicine, University of Montreal, Québec, Canada; 7 Toyama Institute of Health, Toyama, Japan

**Keywords:** *Streptococcus suis*, Genome, Sequence type, Region of difference, Virulence-associated gene

## Abstract

**Background:**

*Streptococcus suis* is a zoonotic pathogen that can cause invasive infections in humans who are in close contact with infected pigs or contaminated pork-derived products. *S. suis* serotype 2 sequence type (ST) 1 strains are mostly associated with meningitis, whereas ST104 strains are mostly recovered from sepsis cases in humans. No data are available for comparison of the ST1 and ST104 strains at the genomic level, particularly concerning virulence-associated genes. Thus, genomic comparison of both STs was performed in this study.

**Methods:**

An ST1 isolate (ID26154) from the cerebrospinal fluid of a patient with meningitis and an ST104 isolate (ID24525) from the blood of a patient with sepsis were subjected to shotgun pyrosequencing using the 454 GS Junior System. Genomic comparison was conducted between the ST1 isolate and the ST104 isolate using the Artemis Comparison Tool (ACT) to identify the region of differences (RDs) between ST1 and ST104.

**Results:**

Fifty-eight RDs were unique to the ST104 genome and were mainly involved in metabolism and cell functional activities, cell wall anchored proteins, bacteriophages and mobile genetic elements, ABC-type transporters, two-component signal transductions, and lantibiotic proteins. Some virulence genes mostly found in ST1 strains were also present in the ST104 genome. Whole-genome comparison is a powerful tool for identifying genomic region differences between different STs of *S. suis* serotype 2, leading to the identification of the molecular basis of virulence involved in the pathogenesis of the infection.

## Introduction

*Streptococcus suis* is a zoonotic pathogen that can cause invasive infections in humans who are in close contact with infected pigs or contaminated pork-derived products. *S. suis* can be classified into 29 serotypes (Okura et al., 2016). Serotype 2 is most frequently detected in infected pigs and humans (Goyette-Desjardins et al., 2014). *S. suis* serotype 2 strains affecting humans include sequence types (STs) 1, 3, 7, 9, 11, 20, 25, 28, 101–105, 107, 126, 134, 144, 146, 233, 298, 337, 379–382, 391–393, 395, and 512–516, based on multilocus sequence typing (MLST) (Goyette-Desjardins et al., 2014; Kerdsin et al., 2018). In addition, most human clinical STs are grouped into limited clonal complexes (CC1, CC20, CC25, CC28, CC104, CC221/234, and CC233/379) (Okura et al., 2016). However, the majority of serotype 2 infections in humans worldwide belong to CC1, especially ST1 (Goyette-Desjardins et al., 2014).

According to our previous reports, human *S. suis* serotype 2-ST1 strains are significantly associated with meningitis. In contrast, those belonging to ST104 are mostly related to non-meningitis cases, particularly sepsis (Kerdsin et al., 2011, 2018). ST1 belongs to CC1, whereas ST104, isolated in Thailand, belongs to CC104 (Kerdsin et al., 2011, 2018). As mentioned above, differences in clinical diseases caused by either ST1 or ST104 may be influenced by genetic backgrounds. No studies have compared the ST1 and ST104 strains at the genomic level, particularly concerning the region of sequence differences (RDs) that may contain virulence-associated genes, pathogenicity islands, and prophages, that may be involved in virulence or the pathogenesis of the infection. Thus, genomic comparison of both STs was performed in this study to understand the pathogenic potential of the strains, especially those ST104 that are more related to sepsis than meningitis.

## Materials and Methods

### Ethical approval

This study used strains obtained from human specimens. The study was reviewed and approved by the Ethics Committees of the Maharaj Nakhon Ratchasima Hospital, Thailand, from where the isolates were obtained (Memorandum no. 8/2564). The Ethics Committees waived the requirement for informed consent as the study did not require any personal patient data and satisfied the conditions of the policy statement on ethical conduct for research involving humans. This study was conducted according to the principles of the Declaration of Helsinki.

### *S. suis* strains and DNA isolation

*S. suis* serotype 2, ST1 (ID26154), and ST104 (ID24525) strains, isolated either from cerebrospinal fluid (CSF) or from a blood sample of human cases of meningitis or sepsis, respectively, were selected for whole genome sequencing. Both strains were cultivated on sheep blood agar at 37 °C and 5% CO_2_. Genomic DNA was extracted using a QIAGEN DNAeasy Blood & Tissue Kit (Qiagen GmbH, Hilden, Germany) according to the manufacturer’s instructions.

### Genome sequencing and analysis

Both STs were subjected to shotgun pyrosequencing using a 454 GS Junior System (Roche). All operations were carried out according to the protocols provided by the manufacturer. The quality of reads was checked using the FASTX-Toolkit package (http://hannonlab.cshl.edu/fastx_toolkit/). The sequencing reads were assembled into contigs using the GS *de novo* Assembler version 2.9 software (Roche, Basel, Switzerland). Genome sequences were submitted to the NCBI Prokaryotic Genome Annotation Pipeline (PGAP v4.12) for annotation.

The comparison of genome sequences was performed using the Artemis Comparison Tool (ACT) to identify the RDs between ST1 and ST104 (Carver et al., 2005). The term RDs refers to the sequences (after genomic comparison) present in the comparable strain but absent in the reference strain. In addition, the following *S. suis* strain sequences were used for comparative genomic analysis: P1/7, BM407, GZ1, SC84, 05ZYH33, 98HAH33, and 89/1591 (Table S1).

All putative open reading frames in the RDs were searched using the ORF finder of the National Center for Biotechnology Information (NCBI; https://www.ncbi.nlm.nih.gov/orffinder/). Protein identification was assigned based on a BLASTP similarity search against the NCBI ‘nr’ (non-redundant protein) database (https://blast.ncbi.nlm.nih.gov/Blast.cgi). Hypothetical proteins were searched for their motifs using the Pfam database (http://pfam.xfam.org/) and MOTIF search tool (https://www.genome.jp/tools/motif/). BLAST Microbial genome, BLASTN, and BLASTX were also used for comparison of nucleotide and protein sequences of those RDs with ≥80% sequence identity and ≥60% coverage (Altschul et al., 1990; States & Gish, 1994; Morgulis et al., 2008; https://blast.ncbi.nlm.nih.gov/Blast.cgi).

The virulence factor database (VFDB) was used to identify virulence genes in the ST1 and ST104 genomes (Liu et al., 2019). Two marker genes (G15: sigma-70 and G20: relaxase mobilization nuclease domain protein) specific to human-associated clade (HAC; Dong et al., 2021) and novel-candidate virulence genes associated with the pathogenic pathotype of *S. suis* (*SSU_RS03100*: hypothetical protein, *SSU_RS09155*: hypothetical protein, and *SSU-RS09525*: RNA-binding protein; Estrada et al., 2022) were used to analyze their presence in the ST1 and ST104 genomes used in the current study based on MyDBFinder 2.0 (https://cge.food.dtu.dk/services/MyDbFinder/).

### Distribution of ST104 RDs in different *S. suis* strains

Of the 58 RDs searched for their similarity to the sequences in the GenBank database based on the BLASTN tool, only 9 were unique to ST104 and they were selected for PCR primer design using the Primer-BLAST tool (https://www.ncbi.nlm.nih.gov/tools/primer-blast/index.cgi?). The primer sequences for PCR screening of ST104-RDs are shown in Table 1. PCR tests targeting the nine selected RDs present in the ST104 strain were performed using the reference strains of the 29 *S. suis* serotypes, *Streptococcus parasuis*, *Streptococcus ruminantium*, *Streptococcus orisratti* (formerly *S. suis* serotypes 20, 22, 26, 32, and 34), and different *S. suis* strains isolated from humans in Thailand (Table 2).

**Table 1:** Primer sequences for screening of RDs in ST104 strains.

Primer names	Sequences (5′ - 3′)	Size of product (bp)
RD2-F	ACC CCT GCA CCT GTC GCT CT	1,857
RD2-R	GGC GGC AAG GGC TGC TTA GT	
RD23-F	AAA GCT GAC GCA GAC CGC GA	840
RD23-R	GGA TGC GGC TTG TGC TGC TG	
RD30-F1*	ATG GCC TTT GAC AGA GAT GGG AGT A	1,005
RD30-R1*	AGC GAT AGC CAG CAC GGA AGG	
RD30-F2*	CCC AGG GAC AAG TCG GTA CA	2,463
RD30-R2*	AGC TAG CAC CCT CTG GAG TC	
RD33-F	TAG TAT TTG GTG AGC CCG TCT TG	1,986
RD33-R	AAA AGG AGC GTA TGT CCC AAC TC	
RD35-F	ATT GGA AAA GGG GGA TTT AGC CAA G	472
RD35-R	TCC CGC TTG ATA ATT CTG GAG CAA	
RD39-F1*	AGA AAA TGG CCT GTT CGG AAT AC	1,586
RD39-R1*	ACG GAG TCG CAT CTA GCA CA	
RD39-F2*	ATG CGA CAA TCA CTC CAG AGC	1,642
RD39-R2*	TAT TCC GAA CAG GCC ATT TTC TCT T	
RD50-F	TGA TTA CTC CTG ATT CTG GAA GCG T	408
RD50-R	TCC TAT GAC TTA CCA TAA CGA CGG T	
RD52-F1*	TCT CCC AAA CGC CAC TCT GAG C	931
RD52-R1*	GGT ATG TCG CTA GCC GTT GGT GC	
RD52-F2*	AAT TGG AGT GCC GTC TGT CG	2,725
RD52-R2*	TAC GAC CGC TTC TGC AAG TG	
RD57-F	GGC AAG CTG CAG CGC TTT TCT GGA G	561
RD57-R	ACG ACC TGC AAG AGT TCG GCA GT	

**Note:**

* Since these RDs were large fragments, two PCR reactions (2 pair primers) were required to efficiently amplify and to cover the whole RDs.

**Table 2:** Distribution of selected 9 RDs of ST104 in *S. suis* strains.

Serotype	CC	ST	RD2	RD23	RD30	RD33	RD35	RD39	RD50	RD52	RD57
2	104	104 (*n* = 100)	+ 100%	+ 100%	+ 100%	+ 100%	+ 100%	+ 100%	+ 100%	+ 100%	+ 76.3%
	233/379	233 (*n* = 14)	+ 100%	–	+ 100%	+ 100%	+ 100%	+ 100%	-	+ 100%	–
	1	1 (*n* = 100)	–	+ 4%	+ 10%	–	+ 4%	–	+ 2%	+ 4%	+ 44%
	28	28 (*n* = 5)	+ 100%	–	–	–	–	+ 80%	–	–	+ 40%
	25	25 (*n* = 14)	+ 100%	–	–	–	–	–	–	–	+ 14.3%
		103 (*n* = 6)	+ 100%	–	–	–	–	–	–	–	–
14	1	105 (*n* = 32)	–	–	+ 6.25%	–	–	–	–	–	+ 40.6%
		127 (*n* = 1)	–	–	–	–	–	–	–	–	+ 100%
		237 (*n* = 1)	–	–	–	–	–	–	–	–	+ 100%
Other (1/2, 1, 3–19, 21, 23–25, 27–31, and *S. suis-*like (formerly serotype 20, 22, 26, and 32–34), (*n* = 44)			+ 22.7%	+ 11.4%	+ 27.3%	+ 13.6%	–	+ 6.8%	+ 9%	–	+ 41%

**Note:**

“+”, percentage of isolates carrying the RDs; “–”, none of the isolates (100%) contains the RDs.

PCR was performed using a total volume of 25 µl containing 1X JumpStart™ REDTaq^®^ ReadyMix™ Reaction Mix (Sigma, St. Louis, MO, USA) and 0.4 µM of each primer (Table 1). The PCR program consisted of an incubation for 3 min at 95 °C, 30 cycles of 30 s at 95 °C, 30 s at 56 °C, and 3 min at 72 °C, and a final extension for 5 min at 72 °C. The PCR products were analyzed by 2% agarose gel electrophoresis in 0.5 × TBE buffer at a constant voltage of 100 Volt for 30 min (Mupid exU system, Takara, Tokyo, Japan). The gels were stained with ethidium bromide and then photographed on an ultraviolet illuminator (GeneGenius Bioimaging System, SynGene, Cambridge, United Kingdom). The sizes of the PCR products were compared with the GeneRuler™ 100 bp Plus DNA ladder (Thermo Fisher Scientific, Waltham, MA, USA) as the molecular size standard. *S. suis* P1/7 was used as a control of all PCR reactions.

## Results and discussion

### General feature of *S. suis* serotype 2 genomes

Sequencing of strain ID26154 (ST1) generated 150,365 reads with an average length of 390.14 bp (range = 40–739 bp), which assembled *de novo* into 36 non-redundant contigs using the GS *de novo* assembler program. The sequencing of strain ID24525 (ST104) generated 148,157 reads with an average length of 386.15 bp (range = 40–624 bp), which assembled *de novo* into 57 non-redundant contigs. The genomes of our ST1 and ST104 strains were 2,029,291 bp and 2,083,364 bp in length, respectively, with GC contents of 41.21 and 41.09, respectively.

The strain ID26154 harbored 1,983 genes with 1,845 coding sequences, three rRNA genes, 42 tRNA genes, and four ncRNA genes. The strain ID24545 harbored 2,086 genes with 1,908 coding sequences, three rRNA genes, 41 tRNA genes, and four ncRNA genes.

### RDs present in ST1 but absent in ST104 genomes

Comparison between the genomes of ST1 and ST104 showed many RDs present in the ST1 genome but absent in the genome of the ST104 strain. The RDs in the ST1 strain were identical to those previously reported for this ST (Table S2) (de Greeff et al., 2011; Zheng et al., 2011). Seventy RDs were identified in the ST1 genomes of the P1/7, BM407, GZ1, and ID26154 strains (Table S2). The size of RDs varied from 213 bp to 17.6 Kb. Of the 70 RDs, some of these genes were present in ST1 only, such as *srtBCD* cluster, *revS*, *epf*, and *rgg*. Six RDs had lower GC contents (<30% GC) than the ST1 (ID26154) chromosome, suggesting putative lateral transferred genes/sequences (Table S2).

Eight RDs (RDs 6, 14, 24, 33, 47, 57, 65, and 66) previously described to be associated with high pathogenicity in CC1, particularly ST1 and ST7, were present in the ST1 strain in the present study (Table S2; Zheng et al., 2011). Only the capsule polysaccharide locus, was present in the ST1 and ST104 strains because both strains belong to serotype 2. This confirmed that the ST1 strain used in the present study should present a virulence potential similar to that of other ST1 and CC1 members. In addition, analysis of the virulence-associated genes showed that many of them were present in the ST1 strain ID26154 (Table 3). Three proposed novel marker genes (*SSU_RS03100*: hypothetical protein, *SSU_RS09155*: hypothetical protein, and *SSU-RS09525*: RNA-binding protein) for predicting the pathogenic pathotype of *S. suis* were also present in our ST1 strain (100% identity), suggesting these are also present in Asian or Eurasian strains (Table 3; Estrada et al., 2022). However, further study needs to be evaluated for these marker genes with *S. suis* strains from different geographical origins.

**Table 3:** Virulence-associated genes in ST1 and ST104 strains in this study.

Virulence genes	Product	ST1 strain ID26154	ST104 strain ID24545
Agglutinin receptor	Agglutinin receptor	**+**	**+**
*arcA*	Arginine deiminase	**+**	**+**
*adcR*	Cation uptake regulator protein	**+**	**+**
*apuA*	Amylopullulanase	**+**	−
*cbpD*	Choline-binding protein	**+**	**+**
*ciaRH*	Two-component regulatory system	**+**	**+**
*covR*	Orphan response regulator	**+**	**+**
*cylR2*	Cytolysin	−	**+**
*ddpvIV*	Dipeptidylpeptidase IV	**+**	**+**
*eno*	Enolase	**+**	**+**
*epf*	Extracellular factor	**+**	−
*fbpS*	Fibronectin-binding protein	**+**	**+**
*feoB*	Fe^2+^ transport protein	**+**	**+**
*fur*	Ferric uptake regulator protein	**+**	**+**
*gapA*	Streptococcal plasmin receptor	**+**	**+**
*gdh*	Glutamate dehydrogenase	**+**	**+**
*glnA*	Glutamine synthetase	**+**	**+**
*hylA*	Hyaluronidase	**+**	**+**
*iga1*	igA1 protease	**+**	**+**
*impdh*	Inosine 5-monophosphate dehydrogenase	**+**	**+**
*luxS*	Quorum sensing	**+**	**+**
*ofs*	Opacity factor	**+**	**+**
*mrp*	Muramidase-released protein	**+**	−
*pavA*	Fibronectin-binding proteins	**+**	**+**
*revS*	Orphan response regulator	**+**	−
*rgg*	Transcription Regulator	**+**	−
*salK/salR*	Two-component signal transduction system	−	−
*sao*	Surface antigen one	**+**	**+**
*sly*	Suilysin	**+**	**+**
*srtA*	Sortase A	**+**	**+**
*srtBCD*	Sortase B, sortase C, sortase D	**+**	−
*srtF*	Sortase F	**+**	**+**
*srtG*	Sortase G	−	**+**
*sspA*	Subtilisin-like protease	**+**	**+**
*tig* or *ropA*	Trigger factor	**+**	**+**
*zmpC*	Zinc metalloproteinase	**+**	**+**
SSU0835-P1/7	ABC-type multidrug transport system	**+**	−
SSU_RS03100	Hypothetical protein	**+**	**+**
SSU_RS09155	Hypothetical protein	**+**	**+**
SSU-RS09525	RNA-binding protein	**+**	**+**

**Note:**

+, present; −, absent.

### RDs present in ST104 but absent in ST1 genomes

Comparison between the genomic sequences of ST104 (ID24525) with the ST1 (ID26154, P1/7, BM407, GZ1), and ST7 (SC84, 98HAH33, 05ZYH33) strains revealed 58 RDs that were present in the ST104 genome; these RDs were absent in the ST1 and ST7 genomes. These RDs contained genes involved in carbohydrate and amino acid metabolism, DNA replication and recombination, metal resistance, cell wall anchored protein with and without C-terminal cell wall sorting signal (CWS) of the LPXTG motif, bacteriophages and other mobile genetic elements, ABC-type transporters, two-component signal transduction, transcription regulators, CRISPR-Cas proteins, restriction endonucleases, and lantibiotic proteins (Table S3). However, 17 RDs in the ST104 genome were also present in the ST25 genome (strain 89-1591) (Table S3).

**Figure 1:**
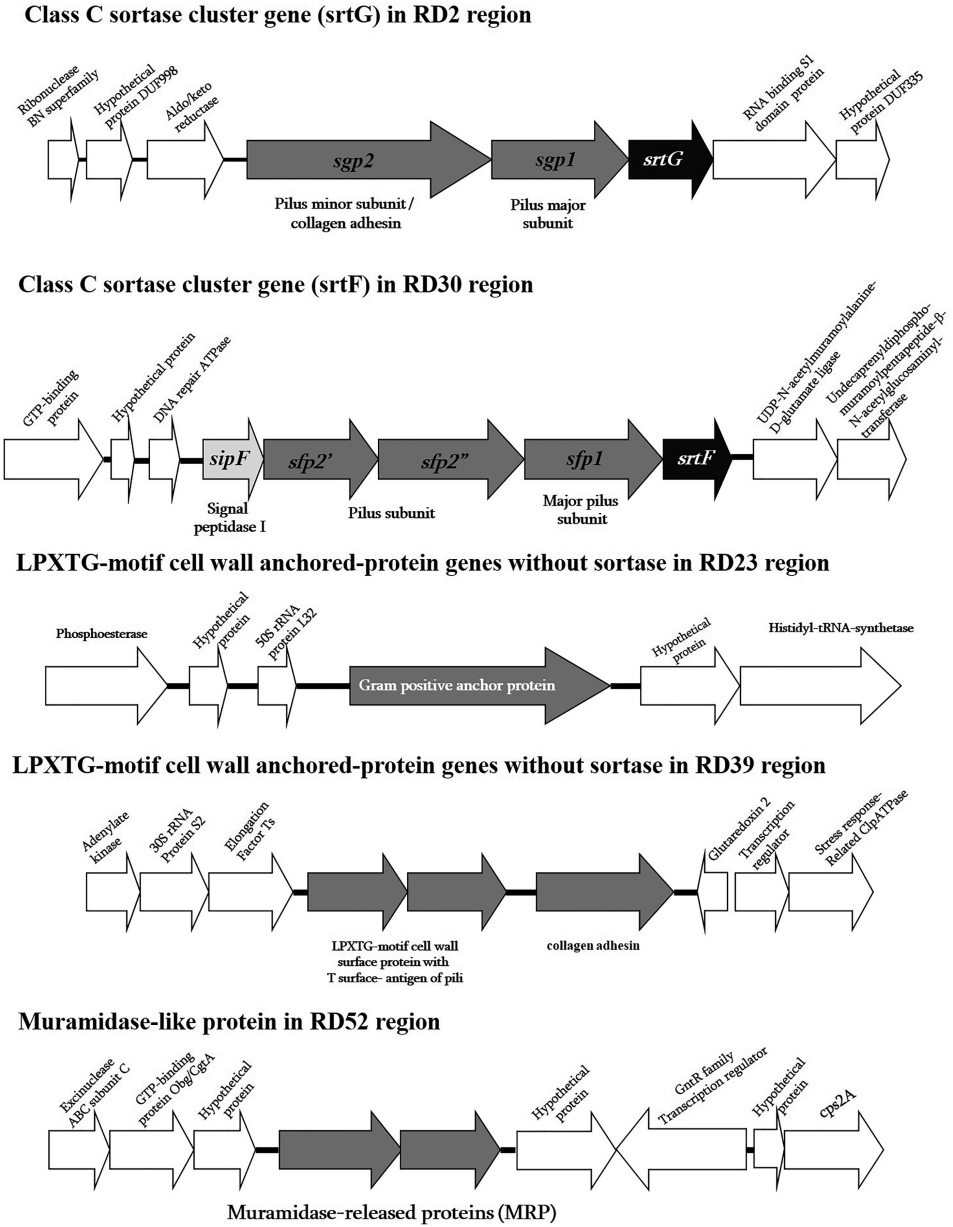
Schematic diagram of surface proteins of *Streptococcus suis* serotype 2 ST104. Schematic diagram of surface proteins with C-terminal cell wall sorting signal gene organization in *Streptococcus suis* serotype 2 ST104.

Among these 58 RDs, the surface or cell wall anchored proteins containing the CWS of ST104 were different from the ST1 and ST7 strains. These included five RDs that were composed of two cluster genes and four individual genes coding for two class C sortase family protein genes in RD2 (*srtG* cluster; 37.57% GC) and RD30 (*srtF* cluster; 39.4% GC), one LPXTG motif cell wall-anchored protein gene in RD23 (37.96% GC), two LPXTG motif cell wall surface protein genes, such as collagen adhesin in RD39 (40.41% GC), and one muramidase-release-like protein gene (*mrp*-like) in RD52 (38.86% GC) (Table S3 and Fig. 1). These surface proteins may be important for human-pathogen interactions, such as adhesion to or invasion of host cells, adhesion to the extracellular matrix, (ECM) and binding to specific immune system components; they may an important role in the initial colonization steps rather than being involved in systemic infection (Navarre & Schneewind, 1999; Schneewind & Missiakas, 2014). More details of these surface proteins in the five RDs are described in the section on surface protein genes in ST104 (below).

In addition to the cell wall surface protein genes described above, ABC-type transporter genes, two-component signal transduction system genes, such as NisK/NisR, and transcription regulator genes might also contribute to ST104 virulence and/or survival in the host (Table S3) (Fittipaldi et al., 2012; Baums & Valentin-Weigand, 2009; Lewis, Ween & McDevitt, 2012; Zheng et al., 2018; Liu et al., 2020). Of these 58 RDs, six had lower GC contents (<30% GC) than the ST104 genome, suggesting putative lateral transferred genes/sequences (Table S3).

### PCR detection of ST104-RDs distribution in different *S. suis* strains

Following the genomic comparison of the ST104 genome with those of the ST1 and ST7 strains, we selected nine candidate RDs (among the 58 RDs) exclusively found in the ST104 strain in this study to evaluate their presence in other *S. suis* strains, by designing PCR primer pairs to amplify each selected RD. As shown in Table 2, only RD23 (37.96% GC) and RD50 (30.3% GC) were present in all ST104 strains but generally absent in ST1 serotype 2 strains; however, they were present in those belonging to other STs. The ST233 (CC233/379) strains contained six RDs similar to ST104, which is closely related to ST104, according to MLST analysis (Kerdsin et al., 2018). Nine selected RDs, except for RD2 and RD57, were mostly present in ST104 (also ST233) strains rather than in other STs of *S. suis* serotypes 2 and 14 (Table 2). However, some CC1 (STs 1, 105, 127, and 237) strains harbored ST104-RDs, as shown in Table 2. Therefore, in certain CC1 strains, either ST104 RDs-homolog or the PCR-targeted region (not whole RDs) may be present.

RD2 containing the *srtG* gene cluster of class C sortase was widely distributed in ST28, ST25, ST103 strains of serotype 2, and in other serotypes (serotypes 1/2, 3, 9, 11, 12, 19, 29, and 30). In addition, a zot-motif protein gene in RD57 was found in ST1, ST28, and ST25 of serotype 2, in ST105, ST127, and ST237 of serotype 14, and in serotypes 3, 7, 9, 16, 17, and 19 (Table 2).

### Surface protein genes in ST104

Surface protein genes encode proteins with LPXTG or related motifs. LPXTG-motif proteins have been involved in the binding of extracellular matrix proteins and adhesion to host cells (Baums & Valentin-Weigand, 2009; Gottschalk et al., 2010). Many proteins with an LPXTG motif have also been suggested to be putative virulence factors, such as the muramidase-released protein (MRP), sortases, surface protein 1 (SAO), and opacity factor (OFS) (Gottschalk et al., 2010).

As previously mentioned, in the ST104 strain analyzed in the present study, five RDs containing genes encoding proteins with an LPXTG motif were described (Fig. 1 and Table S3). When tested using PCR, these genes were present in ST104 strains; however, they were absent in almost all ST1 strains and some other strains (Table 2). These genes were the *srtG* gene cluster (RD2), *srtF* gene cluster (RD30), Gram-positive anchor protein gene (RD23), collagen adhesin and the LPXTG-motif protein with T surface-antigen of pili (RD39), and MRP-like protein (RD52).

The *srtG* gene cluster (RD2) was also found in the ST25, ST28, ST103, and ST233 strains of serotype 2, and in the strains of serotypes 1/2, 3, 9, 11, 12, 19, 29, and 30 (Table 2). However, this cluster was absent in all tested strains of ST1, as shown in Table 2. This gene cluster is composed *of srtG*-encoded sortase, *sgp1*, and *sgp2-*encoded putative pilin subunits (Fig. 1). This structure was similar in *S. suis* strain 89-1591 (ST25) and was predicted to encode putative pili (Takamatsu et al., 2009). In addition, several *S. suis* isolates from humans and diseased pigs had the *srtG* cluster (Takamatsu et al., 2009). The function of the *srtG* cluster-mediated pili is still unknown; however, this gene is expressed at a high level when bacteria are grown at <30 °C (Okura et al., 2011). The surface temperature of different external body parts of pigs ranged from 20 °C to 30 °C when the environmental temperature was approximately 20 °C. This pilus may be involved in the interaction of the bacterium with the host surface components during the first steps of infection (Okura et al., 2011).

The *srtF* gene cluster (RD30) was detected at high frequency in ST104 strains, although it was also found in some ST1 serotype 2 strains and in ST233, serotype 14 ST105, and serotypes 1, 4, 5, 7, 17, 18, 19, and 23 (Table 2). This cluster gene was similar to the *srtG* gene cluster, which encodes a putative pili (Takamatsu et al., 2009). It contains *srtF*, *sfp1*, *sfp2′*, *sfp2*, and *sipF*, which encode for a sortase (*srtF*), putative pilin subunits (*sfp1, sfp2′*, and *sfp2″*), and signal peptidase (*sipF*). We found that the *srtF* gene was identical to that found in ST1 strains (P1/7, BM407, GZ1), whereas *sfp1* was highly similar to that found in other strains (95–100%), such as serotype 1 (accession no. CP002651), serotype 2 strains MNCM50 (ST104; accession no. AB434484) and MNCM21 (ST101; accession no. AB434483), and the unknown serotype DAT299 (ST114; accession no. AB434482) in Genbank. However, *sfp2′* and *sfp2′* were similar to serotype 1 only (accession no. CP002651), with high levels of similarity of 96% and 99%, respectively. The *sfp2′* and *sfp2″* genes in ST104 are separated by nonsense mutations. Accordingly, the proteins Sfp2′ and Sfp2″ showed 42% and 49%, respectively, identity with N-terminal and C-terminal haves, respectively, of the cell wall surface repeat protein of *S. suis* strain 89/1591 (Fig. 1). The *srtF* cluster is essential for the virulence of *Galleria mellonella* when tested in caesarean-derived, colostrum-deprived piglets (Faulds-Pain et al., 2019). Deletion of *srtF* in *S. suis* induces a higher susceptibility to clearance by the innate immune system (Faulds-Pain et al., 2019).

Four individual LPXTG-motif surface protein genes were found in three RDs: RD23, RD39, and RD52. In RD23, a Gram-positive anchor protein gene was identified according to its protein motif using Pfam database identification. This protein has an identity of 72% to the hypothetical protein SSUST1_0363 of *S. suis* serotype 1 (accession no. AER20776), 59% to the hypothetical protein SSUR61_1521 of *S. suis* strain R61 (unknown serotype accession no. EHC02107), 47% to a Gram-positive anchor of *Streptococcus infantis* SK970 (accession no. EGV04462), and 45% to the surface exclusion protein PrgA of *Streptococcus oralis* ATCC 35037 (accession no. ZP07640015). The function of this gene or protein is yet unknown; however, the properties of proteins in the superfamily of Gram-positive anchor proteins suggest that it may be associated with host cell adhesion and invasion (Fischetti, Pancholi & Schneewind, 1990).

RD39 consisted of two LPXTG-motif protein genes, collagen adhesin (or collagen-like protein) and an LPXTG-motif protein containing T-surface antigen of pili based on Pfam database identification. Collagen adhesin is widely present in pathogenic streptococci, including *Streptococcus pyogenes, Streptococcus agalactiae, Streptococcus pneumoniae*, and *Streptococcus equi* (Abranches et al., 2011). The function of collagen adhesin is to bind to ECM, collagen, and laminin, and adherence to human coronary artery endothelial cells, as demonstrated in *Streptococcus mutans* (Abranches et al., 2011). In *S. pyogenes*, the collagen adhesin promotes adhesion and biofilm formation and decreases bacterial killing by neutrophil extracellular traps in tissues (Lukomski et al., 2017). It is also a potential risk factor for hemorrhagic stroke (Nakano et al., 2011). The LPXTG-motif protein containing T-surface antigen of pili has a region similar to the *fctA* gene of *S. pyogenes*, which encodes surface proteins, including fibronectin- and collagen-binding proteins and serological markers known as T antigens, which give rise to pilus-like appendages (Lizano, Luo & Bessen, 2007). In *S. pyogenes*, pili play a role in the adherence and colonization of human tissues (Manetti et al., 2007).

RD52 is composed of two muramidase-release-like protein genes (MRP-like), which show different similarity. The first MRP-like sequence had high similarity to the MRPs of *Gemella haemolysans* ATCC10379, *Granulicatella elegans* ATCC700633, *Streptococcus vestibularis* ATCC49124, and *Streptococcus mitis* NCTC12261 with 53%, 50%, 50%, and 49%, respectively. The second MRP-like sequence was similar to the MRP sequence of *S. suis*, with an identity range of 38–42%. Because of the low similarity with real *S. suis* MRP, the primers used in the PCR tests for *S. suis* MRP could not detect the MRP-like gene in *S. suis* ST104 strains (Silva et al., 2006; Kerdsin et al., 2018).

### Distribution of previously described putative virulence genes in ST104

Our ST104 strain contains genes that may be associated with virulence in the ST1 and ST7 strains, as shown in Table 3 (Fittipaldi et al., 2012; Takamatsu et al., 2009; Zhang et al., 2009; Dumesnil et al., 2018). It carries three proposed novel marker genes (*SSU_RS03100*: hypothetical protein, *SSU_RS09155*: hypothetical protein, and *SSU-RS09525*: RNA-binding protein; Estrada et al., 2022) that predict pathogenicity of *S. suis*, suggesting that this ST104 strain presents a pathogenic pathotype (Table 3; Estrada et al., 2022).

However, five putative virulence genes, *salK/salR*, *srtBCD* gene cluster, *revS*, *rgg*, and *epf*, described as being present in the ST1 or ST7 strains, were absent in ST104 (Fittipaldi et al., 2012; Takamatsu et al., 2009). ST104 strains also lack an additional putative virulence factor, such as SSU0835 (an ABC-type multidrug transport system), which has been described as being involved in the invasion of porcine brain microvascular endothelial cells (Vanier et al., 2009). In addition, *S. suis* can cross the blood-brain barrier, causing meningitis under the action of suilysin, which is cytotoxic to the brain microvascular endothelial cells (Fittipaldi et al., 2012). ST104 strains failed to develop high levels of meningitis in a mouse model due to low or no production of suilysin, due to a negligible level of transcription of the *sly* gene and undetectable *sly* promoter activities (Takeuchi et al., 2014). The lack of putative virulence factors may explain why ST104 caused less meningitis than sepsis.

In addition, Dong et al. (2021) demonstrated the HAC diversified from 1,634 *S. suis* isolates. This HAC is strongly associated with human infections. Among 25 HAC-specific marker genes, two selected genes (G15: sigma-70 and G20: relaxase mobilization nuclease protein) were specific to HAC with 12 training HAC isolates, 21 human isolates, and 10 low-virulence pig isolates, as described elsewhere (Dong et al., 2021). Analysis of these two HAC marker genes in our ST104 revealed that they were absent in the genome. This may indicate that ST104 may not belong to HAC or these two marker genes may not be appropriate for human *S. suis* strains from different geographical regions. However, this strain was isolated from a human patient, suggesting it is pathogenic and it might be related to HAC. More ST104 strains should be extensively analyzed in further studies. On the other hand, our ST1 strain contained these two HAC marker genes (100% identity), suggesting it belonged to HAC.

## Conclusion

This study demonstrated the difference in the genomes of ST1 and ST104 strains of *S. suis* serotype 2 isolated from humans. 58 RDs were unique to the ST104 genome; they were involved in metabolism and cell functional activities, cell wall anchored proteins, bacteriophages and mobile genetic elements, ABC-type transporters, two-component signal transductions, and lantibiotic proteins. Some virulence genes found in ST1 were also present in ST104 cells, suggesting the potential virulence of the latter. Further studies using ST104 strains with *in vitro* and *in vivo* models will help to explain why this ST is mostly involved in sepsis rather than meningitis.

## Supplemental Information

10.7717/peerj.14144/supp-1 Supplemental Information 1 Detail of *Streptococcus suis* strains used in genomic comparison in this study.

10.7717/peerj.14144/supp-2 Supplemental Information 2 Regions of sequence difference (RDs) identified in ST1 and absence in ST104.

10.7717/peerj.14144/supp-3 Supplemental Information 3 Region of sequence difference (RDs) identified in ST104 (ID24525).

## Additional Information and Declarations

### Data Availability

The following information was supplied regarding data availability: The whole-genome shotgun project is available at GenBank/ENA/DDBJ: PRJNA691075; JAGJDV000000000, ID26154 (ST1); JAGJDW000000000, ID24525 (ST104).

## References

[ref-1] Abranches J, Miller JH, Martinez AR, Simpson-Haidaris PJ, Burne RA, Lemos JA (2011). The collagen-binding protein Cnm is required for *Streptococcus mutans* adherence to and intracellular invasion of human coronary artery endothelial cells. Infection and Immunity.

[ref-2] Altschul SF, Gish W, Miller W, Myers EW, Lipman DJ (1990). Basic local alignment search tool. Journal of Molecular Biology.

[ref-3] Baums CG, Valentin-Weigand P (2009). Surface-associated and secreted factors of *Streptococcus suis* in epidemiology, pathogenesis and vaccine development. Animal Health Research Reviews.

[ref-4] Carver TJ, Rutherford KM, Berriman M, Rajandream MA, Barrell BG, Parkhill J (2005). ACT: the artemis comparison tool. Bioinformatics.

[ref-5] de Greeff A, Wisselink HJ, de Bree FM, Schultsz C, Baums CG, Thi HN, Stockhofe-Zurwieden N, Smith HE (2011). Genetic diversity of *Streptococcus suis* isolates as determined by comparative genome hybridization. BMC Microbiology.

[ref-6] Dong X, Chao Y, Zhou Y, Zhou R, Zhang W, Fischetti VA, Wang X, Feng Y, Li J (2021). The global emergence of a novel *Streptococcus suis* clade associated with human infections. EMBO Molecular Medicine.

[ref-7] Dumesnil A, Auger JP, Roy D, Vötsch D, Willenborg M, Valentin-Weigand P, Park PW, Grenier D, Fittipaldi N, Harel J, Gottschalk M (2018). Characterization of the zinc metalloprotease of *Streptococcus suis* serotype 2. Veterinary Research.

[ref-8] Estrada A, Gottschalk M, Gebhart CJ, Marthaler DG (2022). Comparative analysis of *Streptococcus suis* genomes identifies novel candidate virulence-associated genes in North American isolates. Veterinary Research.

[ref-9] Faulds-Pain A, Shaw HA, Terra VS, Kellner S, Brockmeier SL, Wren BW (2019). The *Streptococcus suis* sortases SrtB and SrtF are essential for disease in pigs. Microbiology.

[ref-10] Fischetti VA, Pancholi V, Schneewind O (1990). Conservation of a hexapeptide sequence in the anchor region of surface proteins from gram-positive cocci. Molecular Microbiology.

[ref-11] Fittipaldi N, Segura M, Grenier D, Gottschalk M (2012). Virulence factors involved in the pathogenesis of the infection caused by the swine pathogen and zoonotic agent *Streptococcus suis*. Future Microbiology.

[ref-12] Gottschalk M, Xu J, Calzas C, Segura M (2010). *Streptococcus suis*: a new emerging or an old neglected zoonotic pathogen?. Future Microbiology.

[ref-13] Goyette-Desjardins G, Auger JP, Xu J, Segura M, Gottschalk M (2014). *Streptococcus suis*, an important pig pathogen and emerging zoonotic agent-an update on the worldwide distribution based on serotyping and sequence typing. Emerging Microbes & Infections.

[ref-15] Kerdsin A, Akeda Y, Takeuchi D, Dejsirilert S, Gottschalk M, Oishi K (2018). Genotypic diversity of *Streptococcus suis* strains isolated from humans in Thailand. European Journal of Clinical Microbiology & Infectious Diseases.

[ref-16] Kerdsin A, Dejsirilert S, Puangpatra P, Sripakdee S, Chumla K, Boonkerd N, Polwichai P, Tanimura S, Takeuchi D, Nakayama T, Nakamura S, Akeda Y, Gottschalk M, Sawanpanyalert P, Oishi K (2011). Genotypic profile of Streptococcus suis serotype 2 and clinical features of infection in humans, Thailand. Emerging Infectious Diseases.

[ref-17] Lewis VG, Ween MP, McDevitt CA (2012). The role of ATP-binding cassette transporters in bacterial pathogenicity. Protoplasma.

[ref-18] Liu L, Zhang Q, Xu Z, Chen B, Zhang A, Sun X, Jin M (2020). Screening of virulence-related transcriptional regulators in *Streptococcus suis*. Genes (Basel).

[ref-19] Liu B, Zheng D, Jin Q, Chen L, Yang J (2019). VFDB 2019: a comparative pathogenomic platform with an interactive web interface. Nucleic Acids Research.

[ref-20] Lizano S, Luo F, Bessen DE (2007). Role of streptococcal T antigens in superficial skin infection. Journal of Bacteriology.

[ref-21] Lukomski S, Bachert BA, Squeglia F, Berisio R (2017). Collagen-like proteins of pathogenic streptococci. Molecular Microbiology.

[ref-22] Manetti AG, Zingaretti C, Falugi F, Capo S, Bombaci M, Bagnoli F, Gambellini G, Bensi G, Mora M, Edwards AM, Musser JM, Graviss EA, Telford JL, Grandi G, Margarit I (2007). *Streptococcus pyogenes* pili promote pharyngeal cell adhesion and biofilm formation. Molecular Microbiology.

[ref-23] Morgulis A, Coulouris G, Raytselis Y, Madden TL, Agarwala R, Schäffer AA (2008). Database indexing for production MegaBLAST searches. Bioinformatics.

[ref-24] Nakano K, Hokamura K, Taniguchi N, Wada K, Kudo C, Nomura R, Kojima A, Naka S, Muranaka Y, Thura M, Nakajima A, Masuda K, Nakagawa I, Speziale P, Shimada N, Amano A, Kamisaki Y, Tanaka T, Umemura K, Ooshima T (2011). The collagen-binding protein of *Streptococcus mutans* is involved in haemorrhagic stroke. Nature Communications.

[ref-25] Navarre WW, Schneewind O (1999). Surface proteins of gram-positive bacteria and mechanisms of their targeting to the cell wall envelope. Microbiology and Molecular Biology Reviews.

[ref-26] Okura M, Osaki M, Fittipaldi N, Gottschalk M, Sekizaki T, Takamatsu D (2011). The minor pilin subunit Sgp2 is necessary for assembly of the pilus encoded by the *srtG* cluster of *Streptococcus suis*. Journal of Bacteriology.

[ref-27] Okura M, Osaki M, Nomoto R, Arai S, Osawa R, Sekizaki T, Takamatsu D (2016). Current taxonomical situation of *Streptococcus suis*. Pathogens.

[ref-28] Schneewind O, Missiakas D (2014). Sec-secretion and sortase-mediated anchoring of proteins in Gram-positive bacteria. Biochimica et Biophysica Acta.

[ref-29] Silva LM, Baums CG, Rehm T, Wisselink HJ, Goethe R, Valentin-Weigand P (2006). Virulence-associated gene profiling of *Streptococcus suis* isolates by PCR. Veterinary Microbiology.

[ref-30] States DJ, Gish W (1994). Combined use of sequence similarity and codon bias for coding region identification. Journal of Computational Biology.

[ref-31] Takamatsu D, Nishino H, Ishiji T, Ishii J, Osaki M, Fittipaldi N, Gottschalk M, Tharavichitkul P, Takai S, Sekizaki T (2009). Genetic organization and preferential distribution of putative pilus gene clusters in *Streptococcus suis*. Veterinary Microbiology.

[ref-32] Takeuchi D, Akeda Y, Nakayama T, Kerdsin A, Sano Y, Kanda T, Hamada S, Dejsirilert S, Oishi K (2014). The contribution of suilysin to the pathogenesis of *Streptococcus suis* meningitis. The Journal of Infectious Diseases.

[ref-33] Vanier G, Fittipaldi N, Slater JD, de la Cruz Domínguez-Punaro M, Rycroft AN, Segura M, Maskell DJ, Gottschalk M (2009). New putative virulence factors of *Streptococcus suis* involved in invasion of porcine brain microvascular endothelial cells. Microbial Pathogenesis.

[ref-34] Zhang XH, He KW, Duan ZT, Zhou JM, Yu ZY, Ni YX, Lu CP (2009). Identification and characterization of inosine 5-monophosphate dehydrogenase in *Streptococcus suis* type 2. Microbial Pathogenesis.

[ref-35] Zheng C, Li L, Ge H, Meng H, Li Y, Bei W, Zhou X (2018). Role of two-component regulatory systems in the virulence of *Streptococcus suis*. Microbiological Research.

[ref-36] Zheng X, Zheng H, Lan R, Ye C, Wang Y, Zhang J, Jing H, Chen C, Segura M, Gottschalk M, Xu J (2011). Identification of genes and genomic islands correlated with high pathogenicity in *Streptococcus suis* using whole genome tiling microarrays. PLOS ONE.

