# Supplementary material for: Genomic differences between sequence types 1 and 104 of Streptococcus suis Serotype 2
Source: PeerJ. 2022 Oct 6;10:e14144. doi: 10.7717/peerj.14144 (PMC9548313; doi:10.7717/peerj.14144)
Supplement: Supplemental Information 1 [file peerj-10-14144-s001.docx]

**Table S1.** Detail of *Streptococcus suis* strains used in genomic comparison in this study

| **Strain** | **Serotype** | **Sequence type** | **Source** | **Origin** | **Accession number** |
| --- | --- | --- | --- | --- | --- |
| P1/7 | 2 | ST1 | Diseased pig | UK | NC012925 |
| BM407 | 2 | ST1 | Human | Vietnam | NC012926 |
| GZ1 | 2 | ST1 | Human | China | CP000837 |
| SC84 | 2 | ST7 | Human | China | NC012924 |
| 05ZYH33 | 2 | ST7 | Human | China | CP000407 |
| 98HAH33 | 2 | ST7 | Human | China | CP000408 |
| 89/1591 | 2 | ST25 | Diseased pig | Canada | AAFA00000000 |
