# Supplementary material for: Genomic differences between sequence types 1 and 104 of Streptococcus suis Serotype 2
Source: PeerJ. 2022 Oct 6;10:e14144. doi: 10.7717/peerj.14144 (PMC9548313; doi:10.7717/peerj.14144)
Supplement: Supplemental Information 2 [file peerj-10-14144-s002.docx]

**Table S2.** Regions of sequence difference (RDs) identified in ST1 and absence in ST104

| **RDs of ID26154** | **Position in P1/7** | **Size (bp)** | **% GC content of ID26154** | **ST1** | | | | **Product** | **RDs in de Greeff et al., 2011** | **RDs in Zheng et al., 2011** |
| --- | --- | --- | --- | --- | --- | --- | --- | --- | --- | --- |
|  |  |  |  | **P1/7** | **BM407** | **GZ1** | **ID**  **26154** |  |  |  |
| 1 | 42728-44906 | 2,179 | 29.65 | + | + | + | + | 1. Hypothetical protein |  |  |
| 2 | 46647-47071 | 425 | 30.59 | + | + | + | + | 1. No product |  |  |
| 3 | 55764-56538 | 775 | 33.29 | + | + | + | + | 1. DNA-binding protein or transcriptional regulator |  |  |
| 4 | 97123-98676 | 1,554 | 34.49 | + | + | + | + | 1. Membrane protein  2. Cell division FtsK/SpoIIIE family protein | RD1 | RD4 |
| 5 | 99074-99845 | 772 | 29.79 | + | + | + | + | 1. Hypothetical protein |  |  |
| 6 | 162162-168030 | 5,869 | 41.28 | + | + | + | + | 1. Membrane protein (Pig-X/Pbn1)  2. Hypothetical protein 3. Extracellular factor protein (EF) |  | RD6* |
| 7 | 182488-183037 | 550 | 40.55 | + | + | - | + | 1. Surface-anchored protein |  |  |
| 8 | 200499-200840 | 342 | 29.24 | + | + | + | + | 1. Surface-anchored protein | RD3 | RD8 |
| 9 | 200988-201220 | 233 | 31.76 | + | + | + | + | 1. Surface-anchored protein | RD3 | RD8 |

**Table S2.** Regions of difference (RDs) identified in ST1 and absence in ST104 (continued)

| **RDs of ID26154** | **Position in P1/7** | **Size (bp)** | **% GC content of ID26154** | **ST1** | | | | **Product** | **RDs in de Greeff et al.** | **RDs in Zheng et al.** |
| --- | --- | --- | --- | --- | --- | --- | --- | --- | --- | --- |
|  |  |  |  | **P1/7** | **BM407** | **GZ1** | **ID**  **26154** |  |  |  |
| 10 | 203273-204657 | 1,385 | 30.61 | + | + | + | + | 1. Exported protein  2. Surface-anchored protein or ABC-type transport system or parallel beta-helix repeat domain containing protein | RD3 | RD8 |
| 11 | 230337-233505 | 3,169 | 36.48 | + | + | + | + | 1. Bacteriocin-associated integral membrane protein  2. Transcriptional regulator | RD3 |  |
| 12 | 243398-243673 | 276 | 42.03 | + | + | + | + | 1. Membrane protein |  |  |
| 13 | 250330-250732 | 403 | 45.16 | + | + | + | + | 1. Putative exported protein |  |  |
| 14 | 255906-263619 | 7,714 | 42.04 | + | + | + | + | 1. Hypothetical protein  2. MutT/NUDIX hydrolase family protein  3. Surface-anchored protein, sadP 4. Methyl-accepting chemotaxis protein |  | RD10 |
| 15 | 266562-267030 | 469 | 26.01 | + | + | + | - | 1. Alcohol dehydrogenase |  |  |

**Table S2.** Regions of difference (RDs) identified in ST1 and absence in ST104 (continued)

| **RDs of ID26154** | **Position in P1/7** | **Size (bp)** | **% GC content of ID26154** | **ST1** | | | | **Product** | **RDs in de Greeff et al.** | **RDs in Zheng et al.** |
| --- | --- | --- | --- | --- | --- | --- | --- | --- | --- | --- |
|  |  |  |  | **P1/7** | **BM407** | **GZ1** | **ID**  **26154** |  |  |  |
| 16 | 306320-307134 | 815 | 47.61 | + | + | + | + | 1. Hypothetical protein |  |  |
| 17 | 311626-312247 | 622 | 42.77 | + | + | + | + | 1. Transcription regulator protein | RD4 |  |
| 18 | 313124-314207 | 1,084 | 37.33 | + | + | + | + | 1. Lipase | RD4 |  |
| 19 | 345310-346842 | 1,533 | 32.09 | + | + | + | + | 1. Deoxyguanosinetriphosphate triphosphohydrolase |  |  |
| 20 | 359239-363461 | 4,223 | 30.88 | + | + | + | + | 1. SAM dependent methyltransferase  2. Membrane protein |  |  |
| 21 | 364527-372366 | 7,840 | 38.74 | + | + | + | + | 1. Hypothetical protein  2. ATP-binding protein  3. MerR family transcriptional regulator | RD5 | RD12* |
| 22 | 393232-393496 | 265 | 35.85 | + | + | + | + | 1. Methyltransferase GidB |  |  |

**Table S2.** Regions of difference (RDs) identified in ST1 and absence in ST104 (continued)

| **RDs of ID26154** | **Position in P1/7** | **Size (bp)** | **% GC content of ID26154** | **ST1** | | | | **Product** | **RDs in de Greeff et al.** | **RDs in Zheng et al.** |
| --- | --- | --- | --- | --- | --- | --- | --- | --- | --- | --- |
|  |  |  |  | **P1/7** | **BM407** | **GZ1** | **ID**  **26154** |  |  |  |
| 23 | 443025-451782 | 8,758 | 33.61 | + | + | + | + | 1. DNA-binding protein  2. Transcriptional regulator  3. Hypothetical protein  4. DEAD/DEAH box family helicase  5. Fic protein family  6. Membrane protein | RD6 | RD14 |
| 24 | 457578-463977 | 6,400 | 40.03 | + | + | + | + | 1. Signal peptidase I 2 / major pilus subunit (srtF cluster) | RD7 | RD15* |
| 25 | 580830-584191 | 3,362 | 27.72 | + | + | + | + | 1. Hypothetical protein |  |  |
| 26 | 596984-597538 | 555 | 38.74 | + | + | - | + | 1. Plasmid addiction system, toxin protein  2. Hypothetical protein |  | RD18 |
| 27 | 620417-620745 | 329 | 30.40 | + | + | + | + | 1. Exported protein |  | RD19 |
| 28 | 659493-665217 | 5,725 | 43.14 | + | + | + | + | 1. Type III restriction-modification system, restriction enzyme  2. Modification enzyme of type III restriction-modification system  3. Hypothetical protein | RD11 | RD20 |

**Table S2.** Regions of difference (RDs) identified in ST1 and absence in ST104 (continued)

| **RDs of ID26154** | **Position in P1/7** | **Size (bp)** | **% GC content of ID26154** | **ST1** | | | | **Product** | **RDs in de Greeff et al.** | **RDs in Zheng et al.** |
| --- | --- | --- | --- | --- | --- | --- | --- | --- | --- | --- |
|  |  |  |  | **P1/7** | **BM407** | **GZ1** | **ID**  **26154** |  |  |  |
| 29 | 667742-668357 | 616 | 36.04 | + | + | + | + | 1. Membrane protein |  |  |
| 30 | 674001-682054 | 8,054 | 38.22 | + | + | + | + | 1. Hypothetical protein  2. Type I restriction-modification system, specificity protein (HsdS)  3. Type I restriction-modification system, modification protein (HsdM)  4. Type I restriction-modification system, restriction protein (HsdR) | RD12 | RD21* |
| 31 | 697509-706707 | 9,199 | 41.88 | + | + | + | + | 1. Hypothetical protein  2. Dioxygenase  3. Glycerol uptake facilitator protein  4. Alpha-glycerophosphate oxidase  5. Glycerol kinase  6. DNA-binding protein  7. Pyridine nucleotide-disulphide oxidoreductase | RD14 | RD23 |

**Table S2.** Regions of difference (RDs) identified in ST1 and absence in ST104 (continued)

| **RDs of ID26154** | **Position in P1/7** | **Size (bp)** | **% GC content of ID26154** | **ST1** | | | | **Product** | **RDs in de Greeff et al.** | **RDs in Zheng et al.** |
| --- | --- | --- | --- | --- | --- | --- | --- | --- | --- | --- |
|  |  |  |  | **P1/7** | **BM407** | **GZ1** | **ID**  **26154** |  |  |  |
| 32 | 713043-722097 | 9,055 | 37.66 | + | + | + | + | 1. ABC transporter ATP-binding protein  2. Cobalt ABC transporter permease protein  3. Membrane protein  4. Uridine phosphorylase  5. Crp family transcriptional regulator  6. Thiamine-phosphate pyrophosphorylase  7. Hydroxyethylthiazole kinase  8. Phosphomethylpyrimidine kinase  9. Phosphatase | RD15 | RD24 |
| 33 | 732807-736785 | 3,979 | 40.39 | + | + | + | + | 1. Muramidase-released protein (MRP) |  |  |
| 34 | 852453-854955 | 2,503 | 30.08 | + | + | + | + | 1. Adenylylsulfate kinase-like kinase  2. Abortive infection bacteriophage resistance related protein  3. Hypothetical protein |  | RD29* |
| 35 | 862680-865147 | 2,468 | 33.79 | + | + | + | + | 1. Lantibiotic immunity protein or bacteroiocin operon protein  2. ABC type transporter / lantibiotic transport ATP-binding protein (SSU0835) | RD17 | RD30 |

**Table S2.** Regions of difference (RDs) identified in ST1 and absence in ST104 (continued)

| **RDs of ID26154** | **Position in P1/7** | **Size (bp)** | **% GC content of ID26154** | **ST1** | | | | **Product** | **RDs in de Greeff et al.** | **RDs in Zheng et al.** |
| --- | --- | --- | --- | --- | --- | --- | --- | --- | --- | --- |
|  |  |  |  | **P1/7** | **BM407** | **GZ1** | **ID**  **26154** |  |  |  |
| 36 | 933292-934674 | 1,383 | 37.09 | + | + | + | + | 1. Hypothetical protein  2. DNA-binding protein | RD19 | RD32 |
| 37 | 988570-989025 | 456 | 47.15 | + | + | + | + | 1. Hypothetical protein |  |  |
| 38 | 1009487-1010112 | 626 | 44.25 | + | + | - | + | 1. Acetyltransferase (GNAT) family protein |  |  |
| 39 | 1088782-1089463 | 682 | 35.92 | + | + | + | + | 1. Membrane protein |  | RD37 |
| 40 | 1132083-1132551 | 469 | 34.54 | + | + | + | + | 1. Putative glycosyltransferase |  | RD38 |
| 41 | 1300482-1307619 | 7,138 | 35.37 | + | + | + | + | 1. Type I restriction modification DNA specificity domain protein  2. Type I restriction-modification system, S protein  3. Type I restriction-modification system, M protein (*Eco*EI, M protein) 4. Type I restriction-modification system, R protein (*Eco*EI, R protein) | RD24 | RD40* |
| 42 | 1317438-1318720 | 1,283 | 39.20 | + | + | + | + | 1. Hypothetical protein  2. Exported protein | RD25 | RD42 |

**Table S2.** Regions of difference (RDs) identified in ST1 and absence in ST104 (continued)

| **RDs of ID26154** | **Position in P1/7** | **Size (bp)** | **% GC content of ID26154** | **ST1** | | | | **Product** | **RDs in de Greeff et al.** | **RDs in Zheng et al.** |
| --- | --- | --- | --- | --- | --- | --- | --- | --- | --- | --- |
|  |  |  |  | **P1/7** | **BM407** | **GZ1** | **ID**  **26154** |  |  |  |
| 43 | 1327509-1328103 | 595 | 43.03 | + | + | + | + | 1. DNA binding-membrane protein |  |  |
| 44 | 1363956-1375119 | 11,170 | 36.90 | + | + | + | + | 1. Phage integrase family protein  2. Phage primase  3. Hypothetical protein  4. Phage membrane protein  5. DNA-binding phage protein  6. Phage protein  7. Gp21 protein  8. Phage integrase | RD27 | RD45 |
| 45 | 1422092-1422371 | 280 | 36.79 | + | + | + | + | 1. Hypothetical protein |  |  |
| 46 | 1423309-1424382 | 1,074 | 34.92 | + | + | + | + | 1. Membrane protein  2. PadR family transcriptional regulator |  |  |
| 47 | 1487871-1489068 | 1,198 | 33.64 | + | + | + | + | 1. Serum opacity factor (ofs) | RD30 | RD47 |
| 48 | 1532764-1533083 | 320 | 37.81 | + | + | + | + | 1. Membrane protein OxaA 2 precursur |  |  |
| 49 | 1545320-1545532 | 213 | 41.78 | + | + | + | + | 1. Primosomal protein DnaI |  |  |

**Table S2.** Regions of difference (RDs) identified in ST1 and absence in ST104 (continued)

| **RDs of ID26154** | **Position in P1/7** | **Size (bp)** | **% GC content of ID26154** | **ST1** | | | | **Product** | **RDs in de Greeff et al.** | **RDs in Zheng et al.** |
| --- | --- | --- | --- | --- | --- | --- | --- | --- | --- | --- |
|  |  |  |  | **P1/7** | **BM407** | **GZ1** | **ID**  **26154** |  |  |  |
| 50 | 1560154-1560720 | 567 | 39.15 | + | + | - | + | 1. Membrane protein |  |  |
| 51 | 1573755-1574305 | 551 | 39.20 | + | + | + | + | 1. Membrane protein |  |  |
| 52 | 1630152-1631615 | 1,464 | 44.19 | + | + | + | + | 1. Sucrose-specific phosphotransferase system (PTS), IIABC component |  |  |
| 53 | 1646049-1647318 | 1,270 | 42.13 | + | + | + | + | 1. Membrane protein |  |  |
| 54 | 1707723-1709135 | 1,413 | 38.57 | + | + | + | + | 1. Membrane protein or protein kinase |  |  |
| 55 | 1710587-1710838 | 252 | 40.08 | + | + | + | + | 1. Membrane protein |  |  |
| 56 | 1712348-1716924 | 4,577 | 41.75 | + | + | + | + | 1. leucine-rich protein (Fis-containing protein) 2. Response regulator protein  3. Sensor histidine kinase  4. Integral membrane protein |  | RD51 |

**Table S2.** Regions of difference (RDs) identified in ST1 and absence in ST104 (continued)

| **RDs of ID26154** | **Position in P1/7** | **Size (bp)** | **% GC content of ID26154** | **ST1** | | | | **Product** | **RDs in de Greeff et al.** | **RDs in Zheng et al.** |
| --- | --- | --- | --- | --- | --- | --- | --- | --- | --- | --- |
|  |  |  |  | **P1/7** | **BM407** | **GZ1** | **ID**  **26154** |  |  |  |
| 57 | 1718856-1736433 | 17,578 | 44.02 | + | + | + | + | 1. Extracellular solute-binding protein  2. Binding-protein-dependent transport system membrane protein  3. N-acetyl-beta-D-glucosaminidase  4. Transcriptional regulator/sugar kinase (ROK family protein) 5. Glycosyl hydrolases family protein  6. Hypothetical protein  7. Alpha-1,2-mannosidase  8. Endo-beta-N-acetylglucosaminidase | RD32 | RD51 |
| 58 | 1742084-1747239 | 5,156 | 38.08 | + | + | + | + | 1. Acetyltransferase (GNAT) family protein  2. Outer surface protein  3. Sugar phosphotransferase system (PTS), IIBC component  4. N-acetylmuramic acid 6-phosphate etherase  5. RpiR family regulatory protein | RD33 | RD52 |

**Table S2.** Regions of difference (RDs) identified in ST1 and absence in ST104 (continued)

| **RDs of ID26154** | **Position in P1/7** | **Size (bp)** | **% GC content of ID26154** | **ST1** | | | | **Product** | **RDs in de Greeff et al.** | **RDs in Zheng et al.** |
| --- | --- | --- | --- | --- | --- | --- | --- | --- | --- | --- |
|  |  |  |  | **P1/7** | **BM407** | **GZ1** | **ID**  **26154** |  |  |  |
| 59 | 1787696-1794807 | 6,193 | 46.04 | + | + | + | + | 1. MutT/NUDIX hydrolase family protein  2. Nicotinamide mononucleotide transporter  3. Transcriptional regulator  4. Stress response-related Clp ATPase or ATPases with chaperone activity, ATP-binding subunit  5. CtsR family transcriptional regulator  6. Bacterocin transport accessory protein 7. GNAT family acetyltransferase | RD34 | RD53* &  RD54 |
| 60 | 1815835-1816825 | 991 | 45.11 | + | + | + | + | 1. ABC transporter ATP-binding protein |  |  |
| 61 | 1817434-1820079 | 2,618 | 31.63 | + | + | + | + | 1. Radical SAM superfamily protein  2. Rgg transcriptional regulator |  |  |
| 62 | 1827394-1828113 | 720 | 39.31 | + | + | + | + | 1. Plasmid stabilisation system protein  2. Hypothetical protein |  |  |
| 63 | 1837994-1840495 | 2,482 | 44.68 | + | + | - | + | 1. Homocysteine S-methyltransferase  2. S-methylmethionine permease |  | RD57 |

**Table S2.** Regions of difference (RDs) identified in ST1 and absence in ST104 (continued)

| **RDs of ID26154** | **Position in P1/7** | **Size (bp)** | **% GC content of ID26154** | **ST1** | | | | **Product** | **RDs in de Greeff et al.** | **RDs in Zheng et al.** |
| --- | --- | --- | --- | --- | --- | --- | --- | --- | --- | --- |
|  |  |  |  | **P1/7** | **BM407** | **GZ1** | **ID**  **26154** |  |  |  |
| 64 | 1858223-1859411 | 1,189 | 37.26 | + | + | + | + | 1. Hypothetical protein  2. Membrane protein |  |  |
| 65 | 1903369-1905336 | 1,968 | 35.32 | + | + | + | + | 1. CAAX amino terminal protease family protein  2. RevS regulator | RD36 |  |
| 66 | 1911519-1925793 | 14,275 | 38.14 | + | + | + | + | 1. Sortase (srtB)  2. Sortase (srtC)  3. Sortase (srtD)  4. Accessory pilus subunit  5. Putative pilus subunit protein 6. LPXTG-motif cell wall anchor domain protein (SSU1888-1889) | RD37 | RD60 |
| 67 | 1938176-1939045 | 870 | 42.76 | + | + | + | + | 1. Membrane protein  2. Response regulator of the LytR/AlgR family |  | RD61 |
| 68 | 1945116-1945665 | 550 | 38.00 | + | + | + | + | 1. Putative membrane protein |  |  |

**Table S2.** Regions of difference (RDs) identified in ST1 and absence in ST104 (continued)

| **RDs of ID26154** | **Position in P1/7** | **Size (bp)** | **% GC content of ID26154** | **ST1** | | | | **Product** | **RDs in de Greeff et al.** | **RDs in Zheng et al.** |
| --- | --- | --- | --- | --- | --- | --- | --- | --- | --- | --- |
|  |  |  |  | **P1/7** | **BM407** | **GZ1** | **ID**  **26154** |  |  |  |
| 69 | 1961181-1969758 | 8,578 | 41.57 | + | + | + | + | 1. Beta-glucosidase  2. Membrane protein 3. Response regulator protein of the LytR/AlgR family 4. Sensor histidine kinase | RD38 | RD62 |
| 70 | 1980711-1981620 | 910 | 42.64 | + | + | + | + | 1. MutT/NUDIX hydrolase family protein | RD39 |  |

* = the RDs associated to high pathogenicity with statistically significant

Note: the RDs position of ID26154 was referred according to the position in P1/7 genome.
