# Supplementary material for: Genomic differences between sequence types 1 and 104 of Streptococcus suis Serotype 2
Source: PeerJ. 2022 Oct 6;10:e14144. doi: 10.7717/peerj.14144 (PMC9548313; doi:10.7717/peerj.14144)
Supplement: Supplemental Information 3 [file peerj-10-14144-s003.docx]

**Table S3**. Region of sequence difference (RDs) identified in ST104 (ID24525)

| **RDs** | **Size (bp)** | **% GC content** | **ST1** | **ST7** | **ST25** | **Product** |
| --- | --- | --- | --- | --- | --- | --- |
|  |  |  | **P1/7**  **BM407**  **GZ1**  **ID26154** | **SC84**  **98HAH33**  **05ZYH33** | **89-1591** |  |
| 1 | 12,979 | 38.26 | - | - | - | 1. Phage integrase  2. Prophage antirepressor  3. Phage protein  4. DNA replication protein  5. Phage/plasmid primase |
| 2 | 6,149 | 37.57 | - | - | + | 1. Class C3 sortase family proteins (srtG cluster)  2. LPXTG-motif cell wall anchor domain protein |
| 3 | 820 | 43.78 | - | - | + | 1. Filamentation induced by cAMP protein Fic |
| 4 | 1,511 | 41.96 | - | - | + | 1. PTS system, sucrose-specific IIBC subunit |
| 5 | 19,828 | 41.01 | - | - | + | 1. Glycoside hydrolase family 1 (6-phospho-beta-glucosidase)  2. PTS system lactose-specific EIICB component 3. PTS modulated transcriptional regulator, MtlR family  4. AraC family transcriptional regulator  5. PTS system, beta-glucoside-specific IIABC subunit  6. Transcriptional antiterminator, BglG  7. Cof-like hydrolase |
| 6 | 9,775 | 41.62 | - | - | + (78%) | 1. Integrase family protein  2. Phage associated protein  3. Phage protein  4. Primase  5. Phage/plasmid primase, P4 family |
| 7 | 3,502 | 41.95 | - | - | - | 1. Glutamine amidotransferase, class 1  2. Putative amino acid ABC transporter substrate-binding protein  3. Polar amino acid ABC transporter, inner membrane subunit  4. Glutamine transporter, ATP-binding protein |
| 8 | 2,989 | 39.61 | - | - | + | 1. Hypothetical protein |
| 9 | 1,001 | 34.97 | - | - | - | 1. Hypothetical protein |
| 10 | 718 | 41.64 | - | - | - | 1. Hypothetical protein |
| 11 | 3,090 | 30.94 | - | - | - | 1. ABC transporter-like protein  2. Serine/threonine protein kinase related protein  3. Transcriptional regulator |

**Table S3**. Region of difference (RDs) identified in ST104 (ID24525) (continued)

| **RDs** | **Size (bp)** | **% GC content** | **ST1** | **ST7** | **ST25** | **Product** |
| --- | --- | --- | --- | --- | --- | --- |
|  |  |  | **P1/7**  **BM407**  **GZ1**  **ID26154** | **SC84**  **98HAH33**  **05ZYH33** | **89-1591** |  |
| 12 | 2,989 | 42.09 | - | - | - | 1. Aldo/keto reductase family protein  2. Transposase IS204/IS1001/IS1096/IS1165 family protein |
| 13 | 1,207 | 28.83 | - | - | + | 1. Hypothetical protein |
| 14 | 1,528 | 42.41 | - | - | - | 1. Hypothetical protein |
| 15 | 757 | 46.24 | - | - | + | 1. GCN5-related N-acetyltransferase |
| 16 | 535 | 44.86 | - | - | + | 1. Hypothetical protein |
| 17 | 630 | 27.14 | - | - | - | 1. Hypothetical protein |
| 18 | 1,563 | 37.04 | - | - | - | 1. FtsK/SpoIIIE family |
| 19 | 9,514 | 39.44 | - | - | - | 1. CRISPR-associated helicase  2. CRISPR-associated protein (Cas5_I superfamily)  3. CRISPR-associated protein, Csd1 family  4. CRISPR-associated protein Csd2 (Cas 7_I-C superfamily)  5. CRISPR-associated protein cas4  6. CRISPR-associated protein cas1  7. CRISPR-associated protein cas2 |
| 20 | 466 | 40.99 | - | - | + | 1. Transposase IS4 family protein |
| 21 | 1,181 | 41.57 | - | - | + | 1. Hypothetical protein |
| 22 | 1,048 | 35.11 | - | - | + | 1. Hypothetical protein |
| 23 | 8,191 | 37.96 | - | - | - | 1. Hypothetical protein  2. Gram positive anchored-protein  3. Cadmium resistance protein  4. Cadmium resistance accessory protein CadX  5. Phosphoesterase-like protein  6. Transposase IS4 family protein |
| 24 | 847 | 31.40 | - | - | + | 1. Transcriptional regulator, XRE family |
| 25 | 1,855 | 37.63 | - | - | + | 1. ABC-2 type transporter  2. ABC transporter related protein |
| 26 | 1,024 | 34.77 | - | - | + | 1. Phosphoribosylaminoimidazole carboxylase ATPase subunit |

**Table S3**. Region of difference (RDs) identified in ST104 (ID24525) (continued)

| **RDs** | **Size (bp)** | **% GC content** | **ST1** | **ST7** | **ST25** | **Product** |
| --- | --- | --- | --- | --- | --- | --- |
|  |  |  | **P1/7**  **BM407**  **GZ1**  **ID26154** | **SC84**  **98HAH33**  **05ZYH33** | **89-1591** |  |
| 27 | 3,574 | 34.44 | - | - | + | 1. Type II site-specific deoxyribonuclease  2. DNA adenine methylase |
| 28 | 1,410 | 33.40 | - | - | + | 1. Hypothetical protein |
| 29 | 4,825 | 33.93 | - | - | + | 1. Hypothetical protein  2. Type III restriction protein res subunit |
| 30 | 6,427 | 39.40 | - | - | - | 1. Class C1 sortase family proteins (srtF cluster)  2. LPXTG-motif cell wall anchor domain-containing protein or cell wall ribonucleases G and E  3. Putative pilus subunit proteins |
| 31 | 6,425 | 48.76 | - | - | + | 1. ParB-like protein  2. Plasmid recombination protein  3. Hypothetical protein  4. RNA polymerase sigma factor sigma-70 family  5. Sigma-70 region 4 domain-containing protein |
| 32 | 5,410 | 38.30 | - | - | + | 1. RNA-directed DNA polymerase  2. SNF2 family protein  3. Group II intron reverse transcriptase/maturase |
| 33 | 3,516 | 27.67 | - | - | - | 1. Glycine hydroxymethyltransferase  2. Hypothetical protein  3. Signal Transduction Histidine Kinase (STHK) with GAF sensor |
| 34 | 16,831 | 37.28 | - | - | - | 1. DeoR family transcriptional regulator  2. Rhamnulokinase  3. Arabinose isomerase  4. L-fuculose phosphate aldolase  5. Sugar ABC superfamily ATP binding cassette transporter, sugar-binding protein  6. Sugar ABC superfamily ATP binding cassette transporter, permease protein  7. Sugar ABC superfamily ATP binding cassette transporter, membrane protein  8. Possible alpha-L-fucosidase  9. Alpha-N-acetylgalactosaminidase  10. 6-phosphate glucose kinase  11. Hypothetical protein |
| 35 | 2,862 | 28.83 | - | - | - | 1. Hypothetical protein  2. Putative abortive phage resistance protein |

**Table S3**. Region of difference (RDs) identified in ST104 (ID24525) (continued)

| **RDs** | **Size (bp)** | **% GC content** | **ST1** | **ST7** | **ST25** | **Product** |
| --- | --- | --- | --- | --- | --- | --- |
|  |  |  | **P1/7**  **BM407**  **GZ1**  **ID26154** | **SC84**  **98HAH33**  **05ZYH33** | **89-1591** |  |
| 36 | 5,185 | 40.46 | - | - | - | 1. Similar to beta-lactam resistance factor / aminoacyltransferase femB (FemAB superfamily)  2. Similar to serine/alanine adding enzyme (FemAB superfamily)  3. Putative peptidoglycan branched peptide synthesis protein MurM (FemAB superfamily)  4. Putative HTH-type transcriptional activator mta / transcriptional regulator  5. Hypothetical protein (UPF0158 superfamily)  6. SWIM zinc finger [Streptococcus criceti HS-6] |
| 37 | 1,783 | 43.86 | - | - | - | 1. Resolvase, N-terminal domain protein |
| 38 | 15,517 | 32.20 | - | - | - | 1. Hypothetical protein  2. Tn5252 transposon protein  3. Relaxase  4. Lantibiotic leader peptide processing serine protease  5. Nisin biosynthesis regulatory protein NisR  6. Nisin biosynthesis sensor protein NisK  7. Antibiotic ABC superfamily ATP binding cassette transporter  8. NsuE protein / lantibiotic protection ABC transporter permease  9. Antibiotic ABC superfamily ATP binding cassette transporter permease protein  10. Nisin immunity protein  11. Phage integrase family site-specific recombinase |
| 39 | 7,453 | 40.41 | - | - | - | 1. LPXTG cell wall surface protein  2. Cell wall surface anchor family protein or collagen adhesin  3. Glutaredoxin 2  4. Transcriptional regulator  5. Stress response-related Clp ATPase |
| 40 | 807 | 43.87 | - | - | + | 1. Cytidyltransferase-related domain protein |
| 41 | 1,486 | 39.17 | - | - | - | 1. Hypothetical protein |
| 42 | 1,135 | 37.89 | - | - | + | 1. Hypothetical protein  2. Cytidine/deoxycytidylate deaminase family protein |

**Table S3**. Region of difference (RDs) identified in ST104 (ID24525) (continued)

| **RDs** | **Size (bp)** | **% GC content** | **ST1** | **ST7** | **ST25** | **Product** |
| --- | --- | --- | --- | --- | --- | --- |
|  |  |  | **P1/7**  **BM407**  **GZ1**  **ID26154** | **SC84**  **98HAH33**  **05ZYH33** | **89-1591** |  |
| 43 | 4,109 | 37.11 | - | - | - | 1. Similar to phosphotransferase system protein  2. Phosphotransferase system cellobiose-specific component IIA  3. UbiC transcription regulator-associated or GntR family transcriptional regulator  4. PEP phosphonomutase family protein  5. PTS lactose/cellobiose IIC component |
| 44 | 1,417 | 39.73 | - | - | + | 1. Transposase IS3/IS911 family protein  2. Integrase catalytic region |
| 45 | 3,835 | 33.12 | - | - | + | 1. Hypothetical protein  2. ABC transporter related protein |
| 46 | 877 | 48.23 | - | - | + | 1. Putative transcriptional regulator, PucR family |
| 47 | 9,298 | 38.80 | - | - | + | 1. Tyrosine recombinase xerC / phage integrase family integrase/recombinase  2. DNA binding domain protein, excisionase family  3. hypothetical protein  4. DEAD/DEAH box helicase-like protein |
| 48 | 35,119 | 41.41 | - | - | + | 1. SH3 type-5 domain protein  2. Phage holin, LL-H family  3. Phage minor structural protein  4. Phage protein  5. Putative phage tail protein  6. Phage structural protein  7. Terminase  8. Protein of unknown function DUF1483  9. HNH endonuclease  10. Protein of unknown function DUF1372  11. Single-strand binding protein  12. Protein of unknown function DUF1351  13. Phage recombination protein Bet  14. Phage antirepressor protein  15. Putative phage repressor  16. Transcriptional modulator of MazE/toxin, MazF  17. Integrase family protein  18. Hypothetical protein |
| 49 | 6,472 | 33.48 | - | - | + | 1. Protein of unknown function DUF955  2. Putative transcriptional regulator  3. Hypothetical protein |

**Table S3**. Region of difference (RDs) identified in ST104 (ID24525) (continued)

| **RDs** | **Size (bp)** | **% GC content** | **ST1** | | **ST7** | | **ST25** | **Product** | |  |
| --- | --- | --- | --- | --- | --- | --- | --- | --- | --- | --- |
|  |  |  | **P1/7**  **BM407**  **GZ1**  **ID26154** | | **SC84**  **98HAH33**  **05ZYH33** | | **89-1591** | |  | |
| 50 | 1,000 | 30.30 | - | - | | - | | 1. ABC-type transporter like-protein | |  |
| 51 | 2,995 | 26.41 | - | - | | - | | 1. Hypothetical protein | |  |
| 52 | 5, 275 | 38.86 | - | - | | - | | 1. Muramidase-released like protein (MRP-like protein) | |  |
| 53 | 1,456 | 36.54 | - | - | | + | | 1. Translation initiation factor 2 GTPase | |  |
| 54 | 1,756 | 40.49 | - | - | | + | | 1. ABC transporter related protein  2. Protein of unknown function DUF214 | |  |
| 55 | 1,246 | 41.09 | - | - | | - | | 1. Putative membrane protein  2. Cro/CI family transcriptional regulator | |  |
| 56 | 1,693 | 28.17 | - | - | | + | | 1. Hypothetical protein | |  |
| 57 | 6,976 | 36.10 | - | - | | - | | 1. Replication protein  2. Similar to hypothetical protein pSpnP1_p1  3. Similar to hypothetical protein pSpnP1_p6  4. Hypothetical protein | |  |
| 58 | 1,412 | 39.24 | - | - | | + | | 1. Transposase IS3/IS911 family protein  2. Integrase catalytic region | |  |
